# Obesity-associated outcomes after ACL reconstruction: a propensity-score-matched analysis of the US Nationwide Inpatient Sample 2005–2018

**DOI:** 10.1186/s10195-024-00779-x

**Published:** 2024-07-24

**Authors:** Zhaoyi Fang, Wenxin Liu

**Affiliations:** 1 Biodynamics Laboratory, Department of Orthopedic Surgery, University of Pittsburgh, Pittsburgh, PA USA; 2 Department of Sports Medicine, National Center for Orthopaedics, Shanghai Jiao Tong University Affiliated Sixth People’s Hospital, No. 600 Yishan Road, Shanghai, 200233 China

**Keywords:** Anterior cruciate ligament (ACL), Comorbidity, Nationwide Inpatient Sample (NIS), Obesity, Reconstruction

## Abstract

**Background:**

Anterior cruciate ligament (ACL) injuries are common among physically active individuals, and obesity may increase the risk of such injuries due to factors like biomechanical stress on the knee. We aimed to determine if obesity affects postoperative outcomes after ACL reconstruction.

**Methods:**

Data from adults aged 20 years and older with ACL injuries who underwent inpatient reconstruction from 2005 to 2018 were extracted from the United States (US) Nationwide Inpatient Sample (NIS) database. Patients were divided into two groups based on the presence of co-existing obesity, defined as a body mass index (BMI) ≥ 30 kg/m^2^. Propensity-score matching (PSM) was employed to balance between-group differences. Associations between obesity and concomitant meniscus injury, length of stay (LOS), post-procedural complications, and non-routine discharge were examined using univariate and multivariable logistic and linear regressions.

**Results:**

After PSM, data from 1323 patients (representing 6396 individuals in the US) were analyzed. Of these, 441 (33%) were classified as obese, while 882 (67%) were not obese. After adjustment, obesity was significantly associated with a longer LOS (adjusted beta (aBeta) = 0.32, 95% confidence interval (CI) 0.31–0.321) and an increased likelihood of non-routine discharge (adjusted OR (aOR) = 2.18, 95% CI 1.47–3.22). There were no significant associations between obesity and concomitant meniscus injury (aOR = 1.04, 95% CI 0.81–1.32) or post-procedural complications (aOR = 0.97, 95% CI 0.74–1.27).

**Conclusions:**

In patients undergoing ACL reconstruction in the US, obesity is independently associated with a longer LOS and a higher risk of non-routine discharge. Nevertheless, obesity does not appear to be associated with concomitant meniscus injury or post-procedural complications.

**Supplementary Information:**

The online version contains supplementary material available at 10.1186/s10195-024-00779-x.

## Introduction

Anterior cruciate ligament (ACL) injuries are common in physically active individuals [1]. The overall age- and sex-adjusted annual incidence of ACL tears is reported to be 68.6 per 100,000 person-years [2]. Women have an about 9 times greater risk of ACL injury than men [3]. Although ACL injuries can be treated non-surgically, surgical reconstruction provides maximal stability of the knee [4]. However, the success of ACL surgery can be influenced by several factors, including surgeon experience, sex, ethnicity, graft choice, surgical technique, preoperative muscle strength, and joint range of motion [5, 6].

Obesity is defined as a condition characterized by an excessive accumulation of body fat, typically quantified by a body mass index (BMI) of 30 kg/m^2^ or higher [7]. The global prevalence of obesity has risen markedly over the past several decades [8]. The “obesity epidemic” has raised concerns about the impact of obesity on various health outcomes, including musculoskeletal injuries. Researchers have associated excess body weight with an increased risk of ACL injury [9]. The reasons for this relationship may be attributed to increased biomechanical stress on the knee joint [10], altered movement patterns [10], decreased proprioception [11], or muscular imbalances in obese individuals [12, 13].

Previous research examining the impact of obesity on outcomes following multi-ligament knee injury surgery has reported inconsistent findings [14, 15]. Moreover, there is a relative paucity of studies specifically investigating the influence of obesity on postoperative outcomes after knee ACL reconstruction. Thus, the purpose of this study is to examine the effect of obesity on postoperative outcomes following ACL reconstruction using a nationally representative inpatient database in the United States (US).

## Methods

### Data source

This population-based, retrospective observational study extracted data from the US NIS database, which is the largest continuous inpatient care database in the US and includes data from about 8 million hospital stays each year [16]. The database is administered by the Healthcare Cost and Utilization Project (HCUP) of the US National Institutes of Health (NIH). The patient data consist of primary and secondary diagnoses, primary and secondary procedures, admission and discharge status, patient demographics, projected payment source, hospital stay duration, and hospital characteristics (i.e., bed size, location, teaching status, and hospital area). We initially consider all hospitalized patients for inclusion in the study. The continuously updated, annual NIS database contains patient information from around 1050 hospitals in 44 states, representing a stratified sample of 20% of US community hospitals as defined by the American Hospital Association.

### Ethics statement

All data were obtained through a request to the Online HCUP Central Distributor (available at: https://www.distributor.hcup-us.ahrq.gov/), which administers the database (certificate HCUP-6CVV58M82). This study conforms to the NIS data-use agreement with HCUP. Because this study analyzed secondary data from the NIS database, patients and the public were not involved directly. The study protocol was submitted to the institutional review board (IRB) of our hospital, which exempted the study from IRB approval. Since all data in the NIS database are de-identified, the requirement for informed consent was also waived.

### Study population

Data from patients hospitalized with an ACL injury who received reconstruction surgery between 2005 and 2018 were extracted. Patients with a concomitant diagnosis of posterior cruciate ligament (PCL) disruption or with missing study variables of interest were excluded. All diagnoses and procedures were identified through the International Classification of Diseases, Ninth Revision and Tenth Revision, Clinical Modification (ICD-9-CM, ICD-10-CM) and Procedure Coding System (ICD-9-PCS, ICD-10-PCS), listed in Supplementary Table S1. Patients aged < 20 years, those with a concomitant diagnosis of PCL disruption, and those with missing information were excluded. Patients were then divided into two groups based on their BMI: the non-obese group and the obese group (BMI ≥ 30 kg/m^2^), with obesity status confirmed through corresponding diagnostic codes.

### Outcomes

Primary study outcomes were concomitant meniscus injury, length of hospital stay (LOS), post-procedural complications, and non-routine discharge. LOS was calculated by subtracting the admission date from the discharge date. Post-procedural complications, including venous thromboembolism (VTE), pneumonia, infection, bleeding complication, major blood loss, wound dehiscence, acute kidney injury (AKI), urinary tract infection (UTI), failure of reconstruction (defined as stiffness, effusion, instability, and post-procedural pain), hemarthrosis/joint fistula, post-traumatic osteoarthritis, and any other complication were identified in the patient records. Non-routine discharge was defined as discharge to a long-term care facility.

### Covariates

The patients’ demographic and clinical data were analyzed, including age, sex, insurance status/primary payer, household income, smoking, study year, weekend admission, and Elixhauser comorbidities. The comorbidities of interest were alcohol abuse, anemia, rheumatoid arthritis/collagen vascular diseases, congestive heart failure, chronic pulmonary disease, coagulopathy, depression, uncomplicated diabetes, complicated diabetes, drug abuse, hypertension, hypothyroidism, liver disease, fluid/electrolyte disorders, neurological disorders, paralysis, peripheral vascular disorders, psychoses, pulmonary circulation disorders, renal failure, valvular disease, and weight loss [17]. The codes used to identify the complications and comorbidities are also listed in Supplementary Table 1.

### Statistical analysis

The NIS database covers 20% of the US annual inpatient admissions. Weighted samples (TRENDWT before 2011; DISCWT after 2012), strata (NIS_STRATUM), and clusters (HOSPID) were used to generate national estimates for all analyses. TRENDWT and DISCWT are weights to discharges in the universe, NIS_STRATUM is used to post-stratify hospitals for the calculation of universe and frame weights, and HOSPID is the HCUP hospital identification number. The SURVEY procedure in the SAS software was employed for analyzing sample survey data. Categorical data were presented as the number (*n*) and weighted percentage (%), and continuous data were presented as the mean and standard error (SE). PROC SURVEYFREQ was used for analyzing categorical data, while the PROC SURVEYREG procedure was used for analyzing continuous data. To further balance the baseline characteristics of the comparison groups, the study population was matched using the propensity-score-matching (PSM) method based on age, sex, and study year, with a 1:2 ratio of patients with and without obesity.

Associations between the study variables and the dichotomized outcomes were determined using logistic regression analysis with the PROC SURVEYLOGISTIC statement, and they are presented as odds ratios (ORs) and 95% confidence intervals (CIs). Linear regression analysis was employed to estimate the relation of LOS to the study variables using the PROC SURVEYREG statement, and the results are presented as beta and 95% CI. In cases where significant variables were identified for outcomes, these were included in multivariable regression models for adjustments when comparing differences between the obesity and non-obesity groups. All* p* values were two-sided, and the level of significance was set at 0.05. All statistical analyses were performed using SAS software version 9.4 (SAS Institute Inc., Cary, NC, USA).

## Results

### Patient selection

**Fig. 1:**
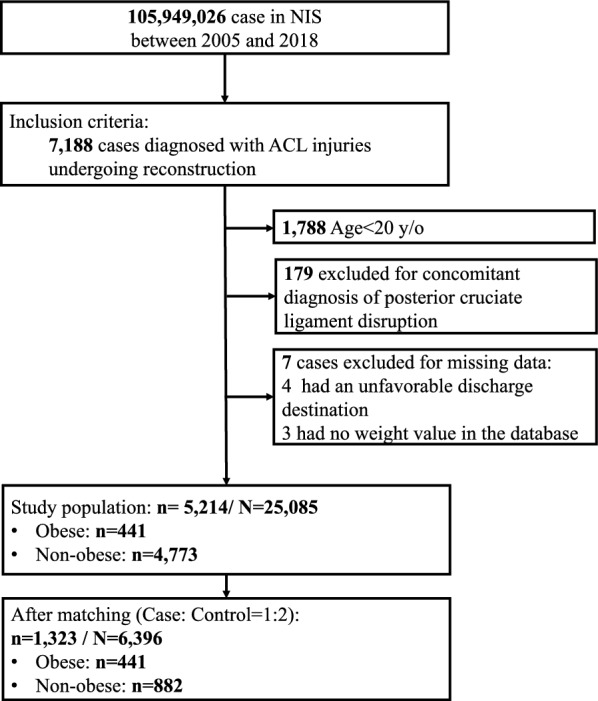
Flow diagram of patient selection and inclusion

A flow diagram of the patient selection and inclusion process is presented in Fig. 1. A total of 7188 patients diagnosed with ACL injuries who underwent inpatient reconstruction were identified in the NIS database between 2005 and 2018. Patients who were under 20 years of age (*n* = 1788) were excluded, along with those with a concomitant diagnosis of PCL disruption (*n* = 179) and those with missing information (*n* = 7), leaving 5,214 patients who met the inclusion criteria for analysis. After 1:2 PSM, 1323 patients remained and were included in the analysis: 441 obese and 882 non-obese patients. This study population can be extrapolated to a population of 6396 individuals in the US after applying the sample weights, as suggested by the NIS dataset.

### Patient characteristics

Patient characteristics before PSM are summarized in Supplementary Table S2. Among the 5214 patients in the overall population, compared to non-obese patients, obese patients were significantly older (38.4 vs. 38.6 years) and included a higher proportion of females (52.9% vs. 53.0%) (all *p* < 0.001).

After PSM, age, sex, and admission years were balanced between the obese and non-obese groups (Table 1). The mean age of the matched study population was 38.5 years, and 47.0% were males. Compared with non-obese patients, obese patients had a higher proportion of smokers (28.0% vs. 20.8%) and more Elixhauser comorbidities, including deficiency anemia (4.8% vs. 2.1%) congestive heart failure (1.6% vs. 0.3%), chronic pulmonary disease (19.2% vs. 8.0%), depression (12.1% vs 6.7%), diabetes (10.9% vs. 4.7% for uncomplicated cases, 2.0% vs. 0.6% for complicated cases), hypertension (34.1% vs. 13.9%), hypothyroidism (6.0% vs. 2.9%), peripheral vascular disorders (1.6% vs. 0.1%), and pulmonary circulation disorders (1.9% vs. 0.7%). Significant differences in the distributions of insurance type and household income were also observed between the two groups (all *p* < 0.05).

**Table 1:** Characteristics of patients after propensity-score matching

Characteristics	All	Obese	Non-obese	*p*
	(*n* = 1323)	(*n* = 441)	(*n* = 882)	
Age, years	38.5 ± 0.291	38.4 ± 0.405	38.6 ± 0.318	0.857
20–29	330 (24.9)	110 (25.0)	220 (24.9)	> 0.999
30–39	418 (31.7)	140 (31.9)	278 (31.6)	
40–49	329 (24.7)	110 (24.7)	219 (24.7)	
50–59	183 (13.9)	60 (13.7)	123 (14.0)	
60+	63 (4.8)	21 (4.8)	42 (4.8)	
Sex				0.974
Male	621 (47.0)	207 (47.1)	414 (47.0)	
Female	702 (53.0)	234 (52.9)	468 (53.0)	
Insurance status/primary payer				** < 0.001**
Medicare/Medicaid	255 (19.6)	112 (25.9)	143 (16.4)	
Private, including HMO	764 (57.9)	216 (49.5)	548 (62.1)	
Self-pay/no charge/other	298 (22.5)	109 (24.7)	189 (21.4)	
Missing	6	4	2	
Household income				**0.009**
Q1	357 (28.3)	115 (27.5)	242 (28.7)	
Q2	322 (25.0)	120 (28.2)	202 (23.4)	
Q3	308 (24.0)	109 (25.8)	199 (23.1)	
Q4	289 (22.7)	79 (18.5)	210 (24.7)	
Missing	46	17	29	
Smoking				** < 0.001**
No	1017 (76.8)	318 (72.0)	699 (79.2)	
Yes	306 (23.2)	123 (28.0)	183 (20.8)	
Study year				0.994
2005–2009	504 (37.1)	168 (37.3)	336 (37.1)	
2010–2015	666 (50.9)	222 (50.7)	444 (51.0)	
2016–2018	153 (12.0)	51 (12.0)	102 (11.9)	
Weekend admission				0.248
No	1213 (91.5)	409 (92.6)	804 (91.0)	
Yes	110 (8.5)	32 (7.4)	78 (9.0)	
Hospital bed size				0.328
Small	218 (16.0)	80 (17.7)	138 (15.1)	
Medium	349 (26.7)	113 (25.9)	236 (27.1)	
Large	746 (57.3)	246 (56.4)	500 (57.8)	
Missing	10	2	8	
Hospital region				0.098
Northeast	225 (17.2)	63 (14.6)	162 (18.5)	
South	284 (21.6)	104 (23.7)	180 (20.6)	
Midwest	461 (34.7)	149 (33.4)	312 (35.3)	
West	353 (26.5)	125 (28.3)	228 (25.6)	
Hospital location/teaching status				**0.016**
Rural	135 (10.3)	34 (7.7)	101 (11.6)	
Urban nonteaching	471 (35.4)	170 (38.2)	301 (33.9)	
Urban teaching	707 (54.3)	235 (54.1)	472 (54.4)	
Missing	10	2	8	
Elixhauser comorbidities				
Alcohol abuse	41 (3.2)	13 (3.0)	28 (3.2)	0.802
Anemia, chronic blood loss	8 (0.6)	3 (0.7)	5 (0.6)	0.837
Anemia, deficiency	39 (3.0)	21 (4.8)	18 (2.1)	**0.004**
Rheumatoid arthritis/collagen vascular diseases	12 (0.9)	7 (1.6)	5 (0.6)	0.079
Congestive heart failure	10 (0.8)	7 (1.6)	3 (0.3)	**0.012**
Chronic pulmonary disease	155 (11.7)	85 (19.2)	70 (8.0)	** < 0.001**
Coagulopathy	17 (1.3)	4 (0.9)	13 (1.5)	**0.024**
Depression	112 (8.5)	53 (12.1)	59 (6.7)	** < 0.001**
Diabetes, uncomplicated	89 (6.8)	48 (10.9)	41 (4.7)	** < 0.001**
Diabetes, complicated	14 (1.1)	9 (2.0)	5 (0.6)	**0.012**
Drug abuse	42 (3.2)	14 (3.3)	28 (3.2)	0.981
Hypertension	274 (20.6)	151 (34.1)	123 (13.9)	** < 0.001**
Hypothyroidism	53 (4.0)	27 (6.0)	26 (2.9)	**0.003**
Liver disease	9 (0.7)	3 (0.7)	6 (0.7)	0.990
Fluid/electrolyte disorders	67 (5.1)	28 (6.3)	39 (4.5)	0.090
Neurological disorders	27 (2.0)	10 (2.2)	17 (1.9)	0.638
Paralysis	4 (0.3)	1 (0.2)	3 (0.4)	0.684
Peripheral vascular disorders	8 (0.6)	7 (1.6)	1 (0.1)	** < 0.001**
Psychoses	46 (3.5)	20 (4.5)	26 (3.0)	0.081
Pulmonary circulation disorders	14 (1.1)	8 (1.9)	6 (0.7)	**0.034**
Renal failure	16 (1.3)	8 (1.9)	8 (0.9)	0.084
Valvular disease	10 (0.8)	6 (1.3)	4 (0.5)	0.070
Weight loss	10 (0.8)	2 (0.5)	8 (0.9)	0.391

Continuous variables are presented as mean ± standard error (SE). Categorical variables are presented as unweighted count (weighted percentage)

*Q* quartile, *HMO* Health Maintenance Organization

*p* values < 0.05 are shown in bold

### Outcomes

A comparison of outcomes of obese and non-obese patients is shown in Table 2. Obese patients had significantly higher percentages of overall post-procedural complications (25.2% vs. 20.3%, *p* = 0.016) and non-routine discharge (16.0% vs. 8.5%, *p* < 0.001) compared to the non-obese patients. Significant differences were observed between the two groups in the rates of VTE (4.0% vs. 1.5%, *p* < 0.002) and failure of reconstruction (12.6% vs. 8.7%, *p* = 0.043).

**Table 2:** Outcomes of patients after propensity-score matching

Outcome	All	Obese	Non-obese	*p*
	(*n* = 1323)	(*n* = 441)	(*n* = 882)	
Concomitant meniscus injury	301 (22.9)	103 (23.3)	198 (22.7)	0.778
Post-procedural complications	290 (21.9)	112 (25.2)	178 (20.3)	**0.016**
VTE	30 (2.3)	17 (4.0)	13 (1.5)	**0.002**
Pneumonia	7 (0.5)	2 (0.4)	5 (0.6)	0.719
Infection	31 (2.4)	9 (2.0)	22 (2.5)	0.540
Bleeding complication	110 (8.4)	37 (8.5)	73 (8.4)	0.943
Wound dehiscence	8 (0.6)	2 (0.5)	6 (0.7)	**0.613**
AKI	10 (0.8)	5 (1.2)	5 (0.6)	0.134
UTI	5 (0.4)	3 (0.7)	2 (0.2)	0.053
Failure of reconstruction	134 (10.0)	57 (12.6)	77 (8.7)	**0.010**
Hemarthrosis/joint fistula	4 (0.3)	2 (0.5)	2 (0.2)	0.505
Post-traumatic osteoarthritis	4 (0.3)	2 (0.5)	2 (0.2)	0.434
LOS^a^, days	3.8 ± 0.162	4.1 ± 0.184	3.7 ± 0.146	0.190
Non-routine discharge^a^	142 (11.0)	69 (16.0)	73 (8.5)	** < 0.001**

Continuous variables are presented as mean ± standard error (SE). Categorical variables are presented as unweighted count (weighted percentage)

*VTE* venous thromboembolism, *AKI* acute kidney disease, *UTI* urinary tract infection, *LOS* length of hospital stay

^a^Excluding patients who died in the hospital

*p* values < 0.05 are shown in bold

### Associations between obesity and outcomes

The relations between obesity and outcomes are summarized in Table 3. Univariate analysis showed that the obese group had a significantly higher risk of post-procedural complications (odds ratio (OR) = 1.32, 95% confidence interval (CI) 1.05–1.67) and non-routine discharge (OR = 2.04, 95% CI 1.52–2.75).

**Table 3:** Associations between obesity and outcomes

Outcome	Obese (BMI ≥ 30 kg/m²)	Univariate		Multivariable	
		OR/beta (95% CI)	*p* value	aOR/aBeta (95% CI)	*p* value
Concomitant meniscus injury^b^	Yes vs. no	1.04 (0.81, 1.32)	0.778	0.97 (0.74, 1.27)	0.828
Post-procedural complications^c^	Yes vs. no	**1.32 (1.05, 1.67)**	**0.017**	1.23 (0.95, 1.60)	0.118
LOS, days^a,d^	Yes vs. no	0.42 (− 0.21, 1.06)	0.190	**0.32 (0.31, 0.32)**	** < 0.001**
Non-routine discharge^a,e^	Yes vs. no	**2.04 (1.52, 2.75)**	** < 0.001**	**2.18 (1.47, 3.22)**	** < 0.001**

*LOS* length of hospital stay

^a^Excluding patients who died in the hospital

^b^Adjusted for age, sex, study year, weekend admission, alcohol abuse, congestive heart failure, hypothyroidism, fluid/electrolyte disorders, peripheral vascular disorders, renal failure, and valvular disease

^c^Adjusted for age, study year, weekend admission, hospital region, hospital location/teaching status, alcohol abuse, anemia (deficiency), coagulopathy, diabetes (complicated), drug abuse, hypertension, hypothyroidism, liver disease, fluid/electrolyte disorders, neurological disorders, paralysis, pulmonary circulation disorders, renal failure, and weight loss

^d^Adjusted for household income, study year, weekend admission, hospital bed size, hospital location/teaching status, alcohol abuse, anemia (chronic blood loss), anemia (deficiency), coagulopathy, diabetes (complicated), drug abuse, hypertension, fluid/electrolyte disorders, pulmonary circulation disorders, renal failure, and weight loss

^e^Adjusted for age, insurance status/primary payer, study year, weekend admission, hospital bed size, hospital region, hospital location/teaching status, alcohol abuse, anemia (deficiency), congestive heart failure, coagulopathy, depression, diabetes (complicated), hypertension, hypothyroidism, liver disease, fluid/electrolyte disorders, paralysis, peripheral vascular disorders, psychoses, pulmonary circulation disorders, renal failure, valvular disease, and weight loss

*p* values < 0.05 are shown in bold

After adjustment, the multivariable regression analysis showed that obesity was significantly associated with a longer LOS (adjusted beta (aBeta) = 0.32, 95% CI 0.31–0.321) and an increased likelihood of non-routine discharge (adjusted OR (aOR) = 2.18, 95% 1.47–3.22). However, no significant associations between obesity and concomitant meniscus injury (aOR = 0.97, 95% CI 0.74–1.27) or the occurrence of post-procedural complications (aOR = 1.23, 95% CI 0.95–1.60) were observed. The full analytic models are documented in Supplementary Tables S3 and S4.

## Discussion

Results of this propensity-score-matched analysis revealed that among US individuals who received operative reconstruction for an ACL injury, obesity was independently associated with a slightly longer LOS and a 2.2-fold greater risk of non-routine discharge compared to those who are not obese.

As obesity prevalence continues to rise globally, researchers are increasingly exploring how weight affects medical and surgical outcomes. A recent study by Alsayed et al. [18] reported that individuals with a BMI ≥ 25 kg/m^2^ were significantly more likely to have a sports-related ACL injury and more likely to have a combined ACL tear than individuals with a lower BMI. Overall, studies in the medical literature have shown that being overweight or obese has adverse effects on the outcomes of ACL repair. Cooper et al. [19] identified 9000 patients in the American College of Surgeons National Surgical Quality Improvement Program database who underwent ACL reconstruction. A BMI of 40 kg/m^2^ or higher was linked to a notably higher risk of 30-day readmission (OR = 3.06) and extended operation times. Similarly, another study confirmed that a higher BMI correlates with longer surgical durations [20]. In patients undergoing outpatient ACL reconstruction, obesity was associated with a significantly higher risk of requiring hospital admission [21]. Another American College of Surgeons database study reported that a higher BMI is associated with the need for an additional surgical procedure at the time of ACL reconstruction [22].

A systematic review and meta-analysis published in 2019 examined the outcomes of ACL reconstruction in overweight and obese patients [23]. The analysis included nine studies, and patients with a BMI > 30 kg/m^2^ had significantly lower International Knee Documentation Committee scores than those with a BMI < 25 kg/m^2^. Patients with a BMI > 25 kg/m^2^ had a significantly higher risk of developing arthritis; however, interestingly, they had a significantly lower risk of needing revision surgery or having a contralateral ACL tear. Another meta-analysis examined risk factors for postoperative surgical-site infection after ACL reconstruction [24]. A number of factors were found to increase the risk of a postoperative infection, including obesity (OR = 1.82, *p* = 0.0005).

It is known that women are more likely to experience an ACL tear than men, a finding confirmed in our study. Wang et al. [25] performed a study of a nationwide database (PearlDiver) to investigate factors affecting ACL tears and outcomes after reconstruction in males vs. females. The authors reported that ACL tears are more common in females, and women are more likely to have them treated by reconstruction. Notably, a BMI > 40 kg/m^2^ was associated with an increased risk of ACL tears in women. A similar database study that included about 3,700 patients with a minimum of 2 years of follow-up found that the significant risk factors for contralateral ACL rupture after primary ACL reconstruction were younger age, female sex, tobacco use, and depression [26]. Notably, obesity and diabetes were not predictors of a contralateral ACL tear.

The higher risk of non-routine discharge among obese patients who undergo operative reconstruction for an ACL injury found in the present study can be attributed to several factors. First, obese individuals often have other metabolic and health conditions like diabetes and heart disease that complicate their recovery process. Secondly, the risks associated with anesthesia, including difficulties in managing airways, are heightened in obese patients, thereby necessitating extended postoperative care in specialized facilities. Furthermore, physical rehabilitation is more challenging due to limited mobility and greater pain, which can slow down recovery and necessitate extended or specialized rehabilitation services.

On the other hand, our findings indicated that obesity was not independently linked with certain expected outcomes, such as concomitant meniscus injury or post-procedural complications. The absence of a significant association between obesity and post-procedural complications could be due to multiple factors. For instance, advances in surgical techniques and improved care protocols may have lessened the impact of obesity on complication rates, especially in healthcare settings with high levels of expertise. Our results do indicate a trend toward increased risks of postoperative complications (aOR = 1.23) in association with obesity, though statistical significance was not achieved; this could suggest that the sample size was too small to adequately detect the effects of obesity. Consequently, there is a need for future prospective studies with larger patient populations to more definitively assess these impacts.

In the present study, children and adolescents were excluded from the study population. Children and adolescents are not exempt from the obesity epidemic, and the mean weights in these groups are increasing, along with those of adults. Burns et al. [27] examined trends in BMI in adults and pediatric patients undergoing ACL reconstruction. They reported that between 2005 and 2015, the percentage of overweight pediatric patients undergoing ACL reconstruction was significantly greater than that of the general population of overweight patients in a single state in the US. Patel et al. [28] reported that after ACL rupture, overweight and obese children had more overall meniscus tears and more irreparable tears than children with a normal BMI. A recent systematic review by Ang et al. [29] found that adolescents with an elevated BMI were more likely to have concomitant meniscal injuries and surgical procedures after an ACL tear than those with a normal BMI.

### Strengths and limitations

The present study has several notable strengths and limitations. Its primary strength lies in its utilization of patient data from a large, nationally representative inpatient database as the analytical sample. This approach has the advantage of assessing a diverse array of cases from multiple medical centers. Furthermore, the inclusion of relatively large sample sizes ensures adequate statistical power, enabling us to assess events that may be rare in single-institution studies. Additionally, the data cover a broad geographic and demographic spectrum, they are nationally representative, and they offer a high degree of generalizability. However, several limitations must be acknowledged. First, the study is inherently limited by its retrospective design, which may entail selection biases. Second, the administrative data used are primarily collected for billing purposes, not for clinical research, which can lead to a lack of detailed clinical information. Also, the reliance on the ICD code system for identifying diagnoses, procedures, and the BMI might introduce coding errors, a common concern in studies employing billing codes. Crucial variables such as operative duration and surgeon experience, both of which are known to influence post-procedural outcomes, were not accessible within the dataset. Furthermore, potential surgical delays due to clinical or organizational factors could bias the results. However, the database lacks complete records of actual surgical dates during the admissions. Finally, because the NIS only reports inpatient data up to the point of discharge, it precludes the examination of long-term outcomes such as complications, reoperations, and functional status beyond the hospital stay. To address these limitations and provide a more comprehensive understanding of the subject matter, further well-designed studies are warranted.

## Conclusions

In the US, among individuals receiving inpatient ACL reconstruction, obesity independently predicts more than double the risk of non-routine discharge. Further prospective study is still needed to determine the impact of obesity on long-term functional recovery in this specific patient subgroup, which could guide future clinical practices and policy decisions.

### Supplementary Information

Supplementary Material 1.

## Data Availability

The datasets analyzed during the current study are available from the corresponding author on reasonable request.

## Abbreviations

ACL: Anterior cruciate ligament
US: United States
NIS: Nationwide Inpatient Sample
PSM: Propensity-score matching
ROM: Range of motion
PCL: Posterior cruciate ligament
