# Supplementary material for: Obesity-associated outcomes after ACL reconstruction: a propensity-score-matched analysis of the US Nationwide Inpatient Sample 2005–2018
Source: J Orthop Traumatol. 2024 Jul 24;25:36. doi: 10.1186/s10195-024-00779-x (PMC11269535; doi:10.1186/s10195-024-00779-x)
Supplement: Supplementary file 1 — Supplementary Material 1. [file 10195_2024_779_MOESM1_ESM.docx]

**Supplementary Table S1. ICD codes used in this study**

| Diagnosis | **ICD-9** | **ICD-10** |
| --- | --- | --- |
| ACL injuries | 844.2, 717.83 | S83.5 |
| Reconstruction surgery | PCS: 81.45 | PCS: 0MQN, 0MQP |
| Obesity | 278.00, 278.01, 278.03, V85.3, V85.4, V85.53, V85.54 | E66.0-E66.2, E66,8, E66.9, Z68.3, Z68.4, Z68.53, Z68.54 |
| VTE | 415, 451-453, 671, 673, 997.2 | I260, I269, I801-803, I808, I809, I820-I823, I828, I829, O082, O223, O871, O882, I81, I82, I80.x |
| Pneumonia | 486, 481, 482.8, 482.3, 484, 115.05, 115.15, 115.95 | A48.1, J12 - J18, B39.2, B39.5, B39.9, A22.1, B25.0, A37.91, B44.0 |
| Infection | 001-139 | L00-L08, B99, T81.43, O86.03 |
|  | 998.5 | T81.4 |
| Bleeding complication | 459, 285.1, 998.1 PCS: 39.98, 99.0 | R58, D62, M96.81, M96.83, M96.84, L76.0, L76.2, L76.3 PCS: 0W380ZZ, 0W383ZZ, 0W384ZZ |
| Wound dehiscence | 998.3, 998.83, 998.89, 998.9 | T81.3, T81.89, T81.9 |
| AKI | 584.x | N17 |
| UTI | 593.3, 996.4, 996.67, 996.69, 996.77, 996.78, 909.3 | N39.0, T84.7-T84.9 |
| Failure of reconstruction (stiffness, effusion, instability, or post-procedural pain) | 718.86, 719.06, 719.46, 719.56, V54.82, V54.9, v54.89, v58.89, 338.18, 338.28 | M25.26, M25.46, M25.56, M25.66, Z51.89, Z47.33, Z47.89, G89.18, G89.28 |
| Hemathrosis/joint fistula | 719.16, 719.86 | M25.06, M25.16 |
| Post-traumatic osteoarthritis | 715.20, 715.26, 715.28 | M17.2-M17.5, M19.92, M19.93 |
| Alcohol abuse | CM_ALCOHOL | F10, E52, G62.1, I42.6, K29.2, K70.0, K70.3, K70.9, T51.x, Z50.2, Z71.4, Z72.1 |
| Anemia, chronic blood loss | CM_BLDLOSS, | D50.0 |
| Anemia, deficiency | CM_ANEMDEF | D50.8, D50.9, D51.x - D53.x |
| Rheumatoid arthritis/collagen vascular diseases | CM_ARTH | L94.0, L94.1, L94.3, M05.x, M06.x, M08.x, M12.0, M12.3, M30.x, M31.0 - M31.3, M32.x - M35.x, M45.x, M46.1, M46.8, M46.9 |
| Congestive heart failure | CM_CHF | I09.9, I11.0, I13.0, I13.2, I25.5, I42.0, I42.5 - I42.9, I43.x, I50.x, P29.0 |
| Chronic pulmonary disease | CM_CHRNLUNG | I27.8, I27.9, J40.x - J47.x, J60.x - J67.x, J68.4, J70.1, J70.3 |
| Coagulopathy | CM_COAG | D65 - D68.x, D69.1, D69.3 - D69.6 |
| Depression | CM_DEPRESS | F20.4, F31.3 - F31.5, F32.x, F33.x, F34.1, F41.2, F43.2 |
| Diabetes, uncomplicated | CM_DM | E10.0, E10.1, E10.9, E11.0, E11.1, E11.9, E12.0, E12.1, E12.9, E13.0, E13.1, E13.9, E14.0, E14.1, E14.9 |
| Diabetes, complicated | CM_DMCX | E10.2 - E10.8, E11.2 - E11.8, E12.2 - E12.8, E13.2 - E13.8, E14.2 - E14.8 |
| Drug abuse | CM_DRUG | F11.x - F16.x, F18.x, F19.x, Z71.5, Z72.2 |
| Hypertension | CM_HTN_C | I10.x I11.x - I13.x, I15.x |
| Hypothyroidism | CM_HYPOTHY | E00.x - E03.x, E89.0 |
| Liver disease | CM_LIVER | B18.x, I85.x, I86.4, I98.2, K70.x, K71.1, K71.3 - K71.5, K71.7, K72.x - K74.x, K76.0, K76.2 - K76.9, Z94.4 |
| Fluid/electrolyte disorders | CM_LYTES | E22.2, E86.x, E87.x |
| Neurological disorders | CM_NEURO | G10.x - G13.x, G20.x - G22.x, G25.4, G25.5, G31.2, G31.8, G31.9, G32.x, G35.x - G37.x, G40.x, G41.x, G93.1, G93.4, R47.0, R56.x |
| Paralysis | CM_PARA | G04.1, G11.4, G80.1, G80.2, G81.x, G82.x, G83.0 - G83.4, G83.9 |
| Peripheral vascular disorders | CM_PERIVASC | I70.x, I71.x, I73.1, I73.8, I73.9, I77.1, I79.0, I79.2, K55.1, K55.8, K55.9, Z95.8, Z95.9 |
| Psychoses | CM_PSYCH | F20.x, F22.x - F25.x, F28.x, F29.x, F30.2, F31.2, F31.5 |
| Pulmonary circulation disorders | CM_PULMCIRC | I26.x, I27.x, I28.0, I28.8, I28.9 |
| Renal failure | CM_RENLFAIL | N17-N19 |
| Non-metastatic cancer | CM_TUMOR | C00.x - C26.x, C30.x - C34.x, C37.x - C41.x, C43.x, C45.x - C58.x, C60.x - C76.x, C97.x |
| Valvular disease | CM_VALVE | A52.0, I05.x - I08.x, I09.1, I09.8, I34.x - I39.x, Q23.0 - Q23.3, Z95.2 - Z95.4 |
| Weight loss | CM_WGHTLOSS | E40.x - E46.x, R63.4, R64 |

ACL, anterior cruciate ligament; PCS, procedure code; VTE, venous thromboembolism; ICD, International Classification of Diseases; AKI, acute kidney injury; UTI, urinary tract infection.

**Supplementary Table S2. Characteristics of patients before propensity score matching**

|  | All | Obese | Non-Obese | p-value |
| --- | --- | --- | --- | --- |
|  | (n=5214) | (n=441) | (n=4773) |  |
| Age, years | 37.2 ± 0.195 | 38.4 ± 0.405 | 37.1 ± 0.203 | **0.029** |
| 20-29 | 1667 (32.0) | 110 (25.0) | 1557 (32.6) | **0.015** |
| 30-39 | 1455 (27.9) | 140 (31.9) | 1315 (27.6) |  |
| 40-49 | 1234 (23.6) | 110 (24.7) | 1124 (23.5) |  |
| 50-59 | 625 (12.0) | 60 (13.7) | 565 (11.9) |  |
| 60+ | 233 (4.5) | 21 (4.8) | 212 (4.5) |  |
| Sex |  |  |  | **<0.001** |
| Male | 3246 (62.8) | 207 (47.1) | 3039 (64.2) |  |
| Female | 1927 (37.2) | 234 (52.9) | 1693 (35.8) |  |
| Insurance status / Primary Payer |  |  |  | **<0.001** |
| Medicare/Medicaid | 832 (16.2) | 112 (25.9) | 720 (15.3) |  |
| Private including HMO | 3144 (60.4) | 216 (49.5) | 2928 (61.4) |  |
| Self-pay/no-charge/other | 1222 (23.4) | 109 (24.7) | 1113 (23.3) |  |
| Missing | 16 | 4 | 12 |  |
| Household income |  |  |  | **0.011** |
| Q1 | 1316 (26.6) | 115 (27.5) | 1201 (26.5) |  |
| Q2 | 1249 (24.9) | 120 (28.2) | 1129 (24.6) |  |
| Q3 | 1192 (23.9) | 109 (25.8) | 1083 (23.7) |  |
| Q4 | 1229 (24.6) | 79 (18.5) | 1150 (25.2) |  |
| Missing | 227 | 17 | 210 |  |
| Smoking |  |  |  | **<0.001** |
| No | 4240 (81.3) | 318 (72.0) | 3922 (82.2) |  |
| Yes | 974 (18.7) | 123 (28.0) | 851 (17.8) |  |
| Study year |  |  |  | **<0.001** |
| 2005-2009 | 3219 (60.9) | 168 (37.3) | 3051 (63.1) |  |
| 2010-2015 | 1736 (34.0) | 222 (50.7) | 1514 (32.4) |  |
| 2016-2018 | 259 (5.2) | 51 (12.0) | 208 (4.5) |  |
| Weekend admission |  |  |  | 0.669 |
| No | 4859 (93.1) | 409 (92.6) | 4450 (93.1) |  |
| Yes | 355 (6.9) | 32 (7.4) | 323 (6.9) |  |
| Hospital bed size |  |  |  | 0.458 |
| Small | 853 (15.9) | 80 (17.7) | 773 (15.7) |  |
| Medium | 1299 (25.1) | 113 (25.9) | 1186 (25.1) |  |
| Large | 3033 (59.0) | 246 (56.4) | 2787 (59.2) |  |
| Missing | 29 | 2 | 27 |  |
| Hospital region |  |  |  | 0.130 |
| Northeast | 929 (18.0) | 63 (14.6) | 866 (18.3) |  |
| South | 1084 (20.9) | 104 (23.7) | 980 (20.7) |  |
| Midwest | 1832 (34.8) | 149 (33.4) | 1683 (34.9) |  |
| West | 1369 (26.3) | 125 (28.3) | 1244 (26.1) |  |
| Hospital location/teaching status |  |  |  | **0.013** |
| Rural | 621 (12.1) | 34 (7.7) | 587 (12.5) |  |
| Urban nonteaching | 1967 (37.4) | 170 (38.2) | 1797 (37.3) |  |
| Urban teaching | 2597 (50.5) | 235 (54.1) | 2362 (50.2) |  |
| Missing | 29 | 2 | 27 |  |
| **Elixhauser comorbidities** |  |  |  |  |
| Alcohol abuse | 128 (2.5) | 13 (3.0) | 115 (2.5) | 0.448 |
| Anemia, chronic blood loss | 20 (0.4) | 3 (0.7) | 17 (0.4) | 0.335 |
| Anemia, deficiency | 115 (2.2) | 21 (4.8) | 94 (2.0) | **<0.001** |
| Rheumatoid arthritis/collagen vascular diseases | 36 (0.7) | 7 (1.6) | 29 (0.6) | **0.015** |
| Congestive heart failure | 20 (0.4) | 7 (1.6) | 13 (0.3) | **<0.001** |
| Chronic pulmonary disease | 437 (8.4) | 85 (19.2) | 352 (7.4) | **<0.001** |
| Coagulopathy | 47 (0.9) | 4 (0.9) | 43 (0.9) | 0.938 |
| Depression | 290 (5.5) | 53 (12.1) | 237 (4.9) | **<0.001** |
| Diabetes, uncomplicated | 220 (4.2) | 48 (10.9) | 172 (3.6) | **<0.001** |
| Diabetes, complicated | 24 (0.5) | 9 (2.0) | 15 (0.3) | **<0.001** |
| Drug abuse | 117 (2.3) | 14 (3.3) | 103 (2.2) | 0.108 |
| Hypertension | 791 (15.2) | 151 (34.1) | 640 (13.4) | **<0.001** |
| Hypothyroidism | 126 (2.4) | 27 (6.0) | 99 (2.1) | **<0.001** |
| Liver disease | 23 (0.5) | 3 (0.7) | 20 (0.4) | 0.425 |
| Fluid/electrolyte disorders | 168 (3.3) | 28 (6.3) | 140 (3.0) | **<0.001** |
| Neurological disorders | 86 (1.6) | 10 (2.2) | 76 (1.6) | 0.270 |
| Paralysis | 13 (0.3) | 1 (0.2) | 12 (0.3) | 0.904 |
| Peripheral vascular disorders | 22 (0.4) | 7 (1.6) | 15 (0.3) | **<0.001** |
| Psychoses | 121 (2.3) | 20 (4.5) | 101 (2.1) | **<0.001** |
| Pulmonary circulation disorders | 20 (0.4) | 8 (1.9) | 12 (0.3) | **<0.001** |
| Renal failure | 30 (0.6) | 8 (1.9) | 22 (0.5) | **<0.001** |
| Non-metastatic cancer | 5 (0.1) | 1 (0.2) | 4 (0.1) | 0.057 |
| Valvular disease | 51 (1.0) | 6 (1.3) | 45 (0.9) | 0.417 |
| Weight loss | 25 (0.5) | 2 (0.5) | 23 (0.5) | 0.954 |

HMO, Health Maintenance Organization.

Continuous variables are presented as mean ± standard error (SE).

Categorical variables are presented as unweighted counts (weighted percentage).

p-value < 0.05 shown in bold.

**Supplementary Table S3. Associations between study variables, concomitant meniscus injury, and post-procedural complications after propensity score matching**

|  | **Concomitant meniscus injury** | |  | **Post-procedural complications** | |
| --- | --- | --- | --- | --- | --- |
|  | **Univariate** | **Multivariable** |  | **Univariate** | **Multivariable** |
|  | **OR (95% CI)** | **aOR (95% CI)** |  | **OR (95% CI)** | **aOR (95% CI)** |
| **Obese vs. non-obese** | 1.04 (0.81, 1.32) | 0.97 (0.74, 1.27) |  | **1.32 (1.05, 1.67)** | 1.23 (0.95, 1.60) |
| **Age, years** |  |  |  |  |  |
| 20-29 | 0.95 (0.71, 1.27) | 0.96 (0.71, 1.31) |  | 1.16 (0.84, 1.62) | 1.08 (0.77, 1.53) |
| 30-39 | 1.04 (0.77, 1.39) | 1.01 (0.74, 1.38) |  | 1.36 (0.97, 1.90) | 1.26 (0.88, 1.80) |
| 40-49 | **1.60 (1.14, 2.26)** | 1.43 (0.99, 2.06) |  | **1.47 (1.01, 2.15)** | 1.17 (0.77, 1.78) |
| 50-59 | 1.55 (0.93, 2.58) | 1.04 (0.58, 1.86) |  | **3.64 (2.29, 5.79)** | **2.14 (1.23, 3.73)** |
| 60+ | **0.75 (0.61, 0.92)** | **0.73 (0.59, 0.90)** |  | 1.01 (0.80, 1.26) |  |
| **Sex (female vs. male)** |  |  |  |  |  |
| Insurance status / Primary Payer | ref |  |  | ref |  |
| Medicare/Medicaid | 1.03 (0.78, 1.37) |  |  | 0.96 (0.73, 1.26) |  |
| Private including HMO | 1.31 (0.95, 1.81) |  |  | 0.89 (0.64, 1.23) |  |
| Self-pay/no-charge/other |  |  |  |  |  |
| Household income | 1.28 (0.94, 1.74) |  |  | 0.93 (0.67, 1.31) |  |
| Q1 | 0.89 (0.64, 1.23) |  |  | 1.18 (0.84, 1.66) |  |
| Q2 | 0.94 (0.68, 1.30) |  |  | 1.25 (0.89, 1.76) |  |
| Q3 | ref |  |  | ref |  |
| Q4 | 0.95 (0.73, 1.24) |  |  | 1.09 (0.84, 1.41) |  |
| **Smoking (yes vs. no)** |  |  |  |  |  |
| **Study year** | ref | ref |  | ref | ref |
| 2005-2009 | 0.99 (0.77, 1.28) | 0.95 (0.73, 1.23) |  | 1.38 (1.07, 1.79) | 1.22 (0.93, 1.60) |
| 2010-2015 | **2.80 (2.05, 3.84)** | 2.38 (1.70, 3.32) |  | 3.11 (2.26, 4.29) | **1.97 (1.38, 2.83)** |
| 2016-2018 | **1.56 (1.08, 2.24)** | 1.21 (0.81, 1.81) |  | 2.12 (1.51, 2.98) | **1.59 (1.08, 2.34)** |
| **Weekend admission** |  |  |  |  |  |
| **Hospital bed size** | ref |  |  | ref |  |
| Small | 1.05 (0.78, 1.43) |  |  | 1.10 (0.83, 1.46) |  |
| Medium | 0.99 (0.75, 1.31) |  |  | 1.25 (0.95, 1.63) |  |
| Large |  |  |  |  |  |
| **Hospital region** | ref |  |  | ref | ref |
| Northeast | **0.69 (0.48, 0.99)** |  |  | **1.62 (1.08, 2.43)** | **1.63 (1.07, 2.47)** |
| South | 0.87 (0.63, 1.21) |  |  | **1.61 (1.12, 2.32)** | **1.52 (1.05, 2.20)** |
| Midwest | 0.80 (0.57, 1.12) |  |  | **1.84 (1.27, 2.67)** | **2.02 (1.38, 2.96)** |
| West | 1.04 (0.81, 1.32) | 0.97 (0.74, 1.27) |  | **1.32 (1.05, 1.67)** | 1.23 (0.95, 1.60) |
| **Hospital location/teaching status** |  |  |  |  |  |
| Rural | ref |  |  | ref | ref |
| Urban nonteaching | 0.95 (0.67, 1.34) |  |  | 1.20 (0.90, 1.60) | 1.03 (0.76, 1.40) |
| Urban teaching | 0.92 (0.66, 1.28) |  |  | **1.72 (1.33, 2.22)** | **1.34 (1.01, 1.79)** |
| Elixhauser comorbidities |  |  |  |  |  |
| Alcohol abuse | **2.23 (1.26, 3.95)** | **1.89 (1.04, 3.43)** |  | **2.15 (1.21, 3.81)** | 1.22 (0.63, 2.36) |
| Anemia, chronic blood loss | 1.10 (0.22, 5.39) |  |  | 0.52 (0.06, 4.16) |  |
| Anemia, deficiency | 0.99 (0.52, 1.91) |  |  | **2.67 (1.47, 4.84)** | 1.70 (0.79, 3.63) |
| Rheumatoid arthritis/collagen vascular diseases | 1.15 (0.31, 4.22) |  |  | 1.11 (0.30, 4.10) |  |
| Congestive heart failure | **3.41 (1.04, 11.23)** | 0.45 (0.12, 1.71) |  | 0.87 (0.27, 2.81) |  |
| Chronic pulmonary disease | 0.72 (0.49, 1.06) |  |  | 1.17 (0.85, 1.61) |  |
| Coagulopathy | 0.98 (0.41, 2.33) |  |  | **5.63 (2.66, 11.93)** | 2.60 (0.93, 7.30) |
| Depression | 1.40 (0.97, 2.00) |  |  | 1.34 (0.91, 1.98) |  |
| Diabetes, uncomplicated | 0.98 (0.66, 1.47) |  |  | 1.16 (0.74, 1.83) |  |
| Diabetes, complicated | 2.41 (0.96, 6.06) |  |  | **3.90 (1.55, 9.81)** | 1.56 (0.55, 4.44) |
| Drug abuse | 1.20 (0.66, 2.16) |  |  | **1.86 (1.06, 3.25)** | 1.32 (0.69, 2.52) |
| Hypertension | 1.15 (0.87, 1.52) |  |  | **1.58 (1.19, 2.09)** | 1.00 (0.71, 1.40) |
| Hypothyroidism | 1.85 (1.09, 3.16) | 1.70 (0.97, 3.00) |  | **1.90 (1.13, 3.18)** | 1.36 (0.75, 2.46) |
| Liver disease | 1.69 (0.50, 5.75) |  |  | **4.43 (1.30, 15.08)** | 2.58 (0.54, 12.36) |
| Fluid/electrolyte disorders | **2.11 (1.36, 3.26)** | 1.55 (0.95, 2.52) |  | **4.85 (3.15, 7.47)** | **2.48 (1.48, 4.14)** |
| Neurological disorders | 1.19 (0.60, 2.36) |  |  | **2.89 (1.45, 5.77)** | 2.41 (1.00, 5.83) |
| Paralysis | 1.10 (0.35, 3.44) |  |  | **0.00 (0.00, 0.00)** |  |
| Peripheral vascular disorders | **3.16 (1.07, 9.31)** | 2.69 (0.81, 8.99) |  | 2.29 (0.82, 6.42) |  |
| Psychoses | 1.09 (0.62, 1.91) |  |  | 0.84 (0.48, 1.49) |  |
| Pulmonary circulation disorders | 0.56 (0.13, 2.43) |  |  | **22.24 (7.27, 68.02)** | **12.06 (3.71, 39.15)** |
| Renal failure | **3.46 (1.52, 7.88)** | 1.97 (0.86, 4.51) |  | **6.07 (2.50, 14.75)** | **3.39 (1.08, 10.65)** |
| Valvular disease | 1.35 (0.39, 4.65) |  |  | 1.64 (0.44, 6.13) |  |
| Weight loss | 1.50 (0.40, 5.64) |  |  | **15.32 (3.25, 72.12)** | 3.94 (0.74, 20.96) |

OR, odds ratio; aOR, adjusted OR; CI, confidence interval; HMO, Health Maintenance Organization; Q, quartile.

NA: The event occurred in 100% of patients in the obese group.

p-value < 0.05 shown in bold.

**Supplementary Table S4. Associations between study variables, LOS, and non-routine discharge after propensity score matching**

|  | **LOS** ^a^**, days** | |  | **Non-routine discharge** ^a^ | |
| --- | --- | --- | --- | --- | --- |
|  | **Univariate** | **Multivariable** |  | **Univariate** | **Multivariable** |
|  | **Beta (95% CI)** | **aBeta (95% CI)** |  | **OR (95% CI)** | **aOR (95% CI)** |
| Obese vs. non-obese |  |  |  |  |  |
| Age, years | **1.51 (1.48, 1.53)** | **0.96 (0.95, 0.97)** |  | 1.45 (0.96, 2.19) |  |
| 20-29 | **0.56 (0.49, 0.62)** | **0.42 (0.39, 0.45)** |  | 1.52 (0.98, 2.37) |  |
| 30-39 | ref | ref |  | ref |  |
| 40-49 | 0.62 (-0.36, 1.61) |  |  | 0.96 (0.68, 1.35) |  |
| 50-59 |  |  |  |  |  |
| 60+ | ref | ref |  | ref | ref |
| Sex (female vs. male) | **1.20 (0.60, 1.80)** | **0.58 (0.55, 0.60)** |  | **1.85 (1.20, 2.85)** | **1.37 (0.84, 2.23)** |
| Insurance status / Primary Payer | **4.72 (3.27, 6.18)** | **2.61 (2.59, 2.64)** |  | **7.04 (4.45, 11.13)** | **4.69 (2.71, 8.09)** |
| Medicare/Medicaid | **6.46 (4.80, 8.13)** | **4.54 (4.47, 4.60)** |  | **3.43 (2.33, 5.06)** | **2.77 (1.63, 4.73)** |
| Private including HMO |  |  |  |  |  |
| Self-pay/no-charge/other | ref | ref |  | ref | ref |
| Household income | 0.55 (-0.13, 1.24) | **0.32 (0.31, 0.33)** |  | **1.80 (1.07, 3.01)** | 2.07 (1.17, 3.66) |
| Q1 | **1.66 (0.95, 2.38)** | **0.96 (0.94, 0.98)** |  | **2.25 (1.39, 3.65)** | **2.33 (1.38, 3.93)** |
| Q2 |  |  |  |  |  |
| Q3 | ref | ref |  | ref | ref |
| Q4 | -0.46 (-1.51, 0.60) | **-0.70 (-1.78, 0.38)** |  | 0.78 (0.50, 1.21) | 0.69 (0.40, 1.19) |
| Smoking (yes vs. no) | 0.29 (-0.75, 1.33) | **0.18 (-1.04, 1.40)** |  | **0.53 (0.34, 0.82)** | **0.41 (0.24, 0.70)** |
| Study year | 0.02 (-1.18, 1.22) | **-0.70 (-1.77, 0.36)** |  | 0.65 (0.41, 1.02) | 0.55 (0.31, 0.97) |
| 2005-2009 |  |  |  |  |  |
| 2010-2015 | **1.51 (1.48, 1.53)** | **0.96 (0.95, 0.97)** |  | 1.45 (0.96, 2.19) |  |
| 2016-2018 | **0.56 (0.55, 0.57)** | **0.15 (0.13, 0.16)** |  | 0.92 (0.58, 1.46) |  |
| Weekend admission | **0.56 (0.49, 0.62)** | **0.42 (0.39, 0.45)** |  | 1.52 (0.98, 2.37) |  |
| Hospital bed size | ref | ref |  | ref |  |
| Small | 0.62 (-0.36, 1.61) |  |  | 0.96 (0.68, 1.35) |  |
| Medium |  |  |  |  |  |
| Large | ref | ref |  | ref | ref |
| Hospital region | **1.20 (0.60, 1.80)** | **0.58 (0.55, 0.60)** |  | **1.85 (1.20, 2.85)** | **1.37 (0.84, 2.23)** |
| Northeast | **4.72 (3.27, 6.18)** | **2.61 (2.59, 2.64)** |  | **7.04 (4.45, 11.13)** | **4.69 (2.71, 8.09)** |
| South | **6.46 (4.80, 8.13)** | **4.54 (4.47, 4.60)** |  | **3.43 (2.33, 5.06)** | **2.77 (1.63, 4.73)** |
| Midwest |  |  |  |  |  |
| West | ref | ref |  | ref | ref |
| Hospital location/teaching status |  |  |  |  |  |
| Rural | ref | ref |  | ref | ref |
| Urban nonteaching | 0.42 (-0.03, 0.87) | **0.32 (0.22, 0.41)** |  | **3.15 (1.50, 6.59)** | 3.02 (1.35, 6.74) |
| Urban teaching | **2.73 (2.02, 3.43)** | **1.51 (1.41, 1.61)** |  | **7.37 (3.68, 14.75)** | **5.96 (2.72, 13.08)** |
| Elixhauser comorbidities |  |  |  |  |  |
| Alcohol abuse | **6.47 (4.03, 8.91)** | **2.83 (2.61, 3.06)** |  | **3.61 (2.01, 6.49)** | **2.70 (1.09, 6.73)** |
| Anemia, chronic blood loss | 4.18 (-0.78, 9.14) |  |  | 2.80 (0.57, 13.63) |  |
| Anemia, deficiency | **1.81 (0.17, 3.45)** | **0.74 (0.73, 0.75)** |  | **4.39 (2.36, 8.14)** | **5.49 (2.33, 12.91)** |
| Rheumatoid arthritis/collagen vascular diseases | 1.50 (-2.26, 5.25) |  |  | 1.47 (0.32, 6.78) |  |
| Congestive heart failure | 1.23 (-1.64, 4.10) |  |  | **8.15 (2.50, 26.51)** | 2.30 (0.64, 8.23) |
| Chronic pulmonary disease | 0.60 (-0.31, 1.52) |  |  | 1.18 (0.76, 1.85) |  |
| Coagulopathy | **7.63 (3.09, 12.18)** | **3.32 (3.31, 3.33)** |  | **4.77 (2.22, 10.23)** | 2.21 (0.78, 6.30) |
| Depression | 0.74 (-0.64, 2.11) |  |  | 1.45 (0.89, 2.36) | 0.74 (0.36, 1.52) |
| Diabetes, uncomplicated | -0.22 (-1.19, 0.75) |  |  | 1.31 (0.76, 2.26) |  |
| Diabetes, complicated | **2.99 (0.80, 5.18)** | **0.25 (0.23, 0.27)** |  | **11.36 (4.66, 27.68)** | 3.32 (1.00, 11.06) |
| Drug abuse | **5.88 (0.40, 11.36)** | **3.73 (3.70, 3.77)** |  | 1.74 (0.84, 3.58) |  |
| Hypertension | **1.20 (0.22, 2.18)** | **0.36 (0.36, 0.37)** |  | **2.26 (1.63, 3.15)** | 1.34 (0.80, 2.22) |
| Hypothyroidism | 0.28 (-1.17, 1.74) |  |  | **2.60 (1.45, 4.66)** | 1.34 (0.66, 2.70) |
| Liver disease | 4.49 (-0.26, 9.24) |  |  | **4.18 (1.07, 16.34)** | 2.54 (0.69, 9.29) |
| Fluid/electrolyte disorders | **7.59 (5.19, 9.99)** | **4.46 (4.44, 4.47)** |  | **4.75 (2.96, 7.62)** | 1.81 (0.89, 3.70) |
| Neurological disorders | 3.36 (-0.49, 7.22) |  |  | 0.68 (0.16, 2.84) |  |
| Paralysis | -0.71 (-2.88, 1.46) |  |  | **7.70 (1.35, 44.11)** | **29.36 (7.00, 123.18)** |
| Peripheral vascular disorders | 1.88 (-1.56, 5.31) |  |  | **4.51 (1.47, 13.90)** | 1.31 (0.18, 9.27) |
| Psychoses | 4.17 (-0.98, 9.31) |  |  | **2.16 (1.20, 3.90)** | **2.46 (1.10, 5.49)** |
| Pulmonary circulation disorders | **7.27 (2.62, 11.91)** | **4.09 (4.09, 4.10)** |  | **3.32 (1.19, 9.21)** | 0.92 (0.23, 3.67) |
| Renal failure | **4.11 (1.03, 7.19)** | **2.41 (2.40, 2.42)** |  | **2.76 (1.17, 6.51)** | 0.44 (0.11, 1.71) |
| Valvular disease | 3.08 (-3.96, 10.12) |  |  | **0.00 (0.00, 0.00)** | **0.00 (0.00, 0.00)** |
| Weight loss | **15.68 (10.96, 20.39)** | **9.58 (9.54, 9.62)** |  | **6.72 (1.96, 22.99)** | 1.87 (0.23, 15.28) |

OR, odds ratio; aOR, adjusted OR; CI, confidence interval; LOS, length of hospital stays; HMO, Health Maintenance Organization; Q, quartile.

p-value < 0.05 shown in bold.

^a^ Excluding patients who died in the hospital.
